# Signal Processing for Neural Spike Trains

**DOI:** 10.1155/2010/698751

**Published:** 2010-05-05

**Authors:** Theodore W. Berger, Zhe (Sage) Chen, Andrzej Cichocki, Karim G. Oweiss, Rodrigo Quian Quiroga, Nitish V. Thakor

**Affiliations:** ^1^Department of Biomedical Engineering, Viterbi School of Engineering, University of Southern California, Los Angeles, CA 90089, USA; ^2^Neuroscience Statistics Research Laboratory, Massachusetts Institute of Technology, Cambridge, MA 02139, USA; ^3^Laboratory for Advanced Brain Signal Processing, RIKEN Brain Science Institute, Wako-shi, Saitama 351-0198, Japan; ^4^Department of Electrical and Computer Engineering, Michigan State University, MI 48824, USA; ^5^Department of Engineering, University of Leicester, Leicester LE1 7RH, UK; ^6^School of Medicine, Johns Hopkins University, Baltimore, MD 21205, USA

Signal processing and statistics have been playing a pivotal role in computational neuroscience and neural engineering research. Advances in technology have enabled us to simultaneously record extracellular neuronal signals through hundreds of electrode arrays, from which spike trains and local field potentials (LFPs) measurements are obtained. Neural spikes, also known as action potentials, are 0 and 1 observations recorded and subsequently extracted from the output of spiking neurons. To obtain a discrete form of neural spike trains, from either single or multiunits, raw neuronal signals have to be processed properly by some operations, such as filtering, thresholding, detection, and sorting. In the past, signal processing theories and algorithms have been applied for neural spike train analysis. The main objective of this special issue of *Computational Intelligence and Neuroscience* (CIN) is to provide a forum for revisiting some fundamental and important issues with newly developed signal-processing tools. 

This special issue includes 10 contributions that cover a wide range of signal-processing techniques and approaches for neural spike train analysis, including detection, sorting, encoding, and decoding. Loosely, these 10 papers may be classified into three categories. 

*(1) Recording and Analysis of Neuronal Spikes from Single Neuron*. The first paper “Quantitative estimation of the non-stationary behavior of neural spontaneous activity” by Destro-Filho et al. describes a quantitative approach to estimate the nonstationary behavior of neuronal spontaneous activity. The authors use a detrended fluctuation analysis (DFA) algorithm to estimate the “stationary time,” and apply the analysis to interspike interval time series to retrieve information from neural signals. These quantitative measures may help to characterize and reveal nonstationary nature underlying important physiological phenomena. The paper “Stability of neural firing in the trigeminal nuclei under mechanical whisker stimulation” by Makarov et al. describes a method that uses the time evolution of ridges in the wavelet transform spectrum of spike trains to quantify the dynamical stability of neuronal responses to a sensory stimulus. The nonstationary nature of the neuronal responses to tactile whisker stimulation is studied therein, showing that neurons from rat trigeminal nuclei can perform as stimulus frequency filter and adapt their neural coding scheme according to the stimulus characteristic. The paper “Spike sorting of muscle spindle afferent nerve activity recorded with thin-film intrafascicular electrodes” by Djilas et al. describes a neuronal spike detection and classification scheme for separating activity of primary and secondary afferents of muscle spindle activity. The detection algorithm, based on a multiscale continuous wavelet transform, is shown to outperform the standard threshold detection scheme especially under a low signal-to-noise ratio condition. Because of its strength of isolating activity related to the muscle length, this algorithm has a potential application for the closed-loop functional electrical stimulation (FES) system with natural sensory feedback. The paper “State-space algorithms for estimating spike rate functions” by Smith et al. addresses a fundamental question of determining changes in activity in neural firing, and the authors propose a state-space model to estimate the spike rate function and compare their approach with the established Bayesian adaptive regression splines (BARSs) algorithm and a cubic spline smoothing algorithm. Their algorithm is computationally efficient and is practically applicable to a wide range of neurophysiological data. 

*(2) Signal Processing Algorithms for Analysis of Multiple Spike Trains from Neuronal Ensembles*. The paper “Consistent recovery of sensory stimuli encoded with MIMO neural circuits” by Lazar and Pnevmatikakis describes a method for reconstructing finite-energy stimuli encoded with a population of spiking leaky integrate-and-fire neurons and demonstrates how the algorithm can be applied to stimuli encoded with recurrent or multiinput multioutput (MIMO) neural circuits. The paper “Multivariate auto-regressive modeling and Granger causality analysis of multiple spike trains” by Krumin and Shoham describes a new method for estimating the Granger causality of multiple spike trains, which generalizes the causality concept from continuous- to discrete-valued measurements. The new framework allows performing Granger causality analysis to extract the directed information flow pattern amongst neuronal ensembles. The paper “Efficient identification of assembly neurons within massively parallel spike trains” by Berger et al. describes a method for efficient identification of complexity patterns amongst assembly neurons using parallel spike train recordings. The authors propose a number of test statistics and use surrogate data to test the validity of their approach, which allows them to a refined analysis of the detailed high-order correlation structure with desirably reduced computational burden. 

*(3) Signal Processing Applications*. The paper “Development and validation of a spike detection and classification algorithm aimed at implementation on hardware devices” by Biffi et al. proposes a practical spike detection and classification algorithm aiming at FPGA (field-programmable gay array) hardware implementation, which requires online and real-time analysis, and efficient memory usage and data transmission rate. The proposed method using an amplitude-threshold detection and PCA-based hierarchical classifier is evaluated on simulated data with demonstrated efficiency. The paper “Ensemble fractional sensitivity: a quantitative approach to neuron selection for decoding motor tasks” by Singhal et al. proposes an ensemble fractional sensitivity metric for selecting population of neurons, which leads to an improvement in neural decoding accuracy of motor tasks. This approach has potential benefits in designing BCIs where high decoding accuracy is required given limited number of electrodes or training data. The last paper “Decoupling action potential bias from cortical local field potentials” by David et al. aims to solve a practical issue frequently occurring in experiments, where the LFP signals are corrupted by the bias contributed from action potentials induced by small subset of isolated neurons. The authors propose a linear filtering method to remove the features correlated with spike events from LFP recordings, and they show that removing spike-correlated components can affect the auditory tuning of the LFP in real data analysis. 

In summary, this special issue covers practical signal processing issues and new developments for neural spike train analysis in the growing field of computational neuroscience. We hope that the readership of CIN could appreciate this special issue and feel the excitement while reading these articles. That being said, we are reminded that there remain many open research questions that call for further development of signal-processing tools and applications. 

Lastly, the guest editors of this special issue are very grateful to all contributed authors, reviewers, and editorial staffs who had all put tremendous effort to make this issue a reality. 

*Theodore W. Berger* *Zhe (Sage) Chen* *Andrzej Cichocki* *Karim G. Oweiss* *Rodrigo Quian Quiroga* *Nitish V. Thakor*

